# Synthesis and crystal structure of the adduct between 2-pyridyl­selenyl chloride and isobutyro­nitrile

**DOI:** 10.1107/S2056989024000938

**Published:** 2024-02-06

**Authors:** Ayalew W. Temesgen, Alexander A. Sapronov, Alexey S. Kubasov, Alexander S. Novikov, Tuan Anh Le, Alexander G. Tskhovrebov

**Affiliations:** a Department of Chemistry, College of Natural and Computational Science, University of Gondar, Gondar 196, Ethiopia; b Peoples’ Friendship University of Russia, 6 Miklukho-Maklaya Street, Moscow, 117198, Russian Federation; c Kurnakov Institute of General and Inorganic Chemistry, Russian Academy of Sciences, Leninsky Prosp. 31, 119071 Moscow, Russian Federation; d Institute of Chemistry, Saint Petersburg State University, Universitetskaya Nab. 7/9, 199034 Saint Petersburg, Russian Federation; e University of Science, Vietnam National University, Hanoi, 334 Nguyen Trai, Thanh Xuan, Hanoi, 100000, Vietnam; Universidade de Sâo Paulo, Brazil

**Keywords:** crystal structure, chalcogen-hydrogen bonding, 1,2,4-seleno­diazole

## Abstract

The reaction between 2-pyridyl­selenenyl chloride and isobutyro­nitrile results in the formation of the corresponding cationic pyridinium-fused 1,2,4-seleno­diazole, namely, 3-(propan-2-yl)-1,2,4-[1,2,4]selena­diazolo[4,5-a]pyridin-4-ylium chloride, C_9_H_11_N_2_Se^+^·Cl^−^, in high yield (89%). The structure of the compound, established by means of single-crystal X-ray analysis at 100 K, has monoclinic (*P*2_1_/*c*) symmetry and revealed the presence of bifurcated chalcogen-hydrogen bonding Se⋯Cl^−^⋯H—Cl, and these non-covalent contacts were analysed by DFT calculations followed by a topological analysis of the electron-density distribution (ωB97XD/6-311++G** level of theory).

## Abstract 2

The reaction between 2-pyridyl­selenenyl chloride and isobutyro­nitrile results in the formation of the corresponding cationic pyridinium-fused 1,2,4-seleno­diazole, namely, 3-(propan-2-yl)-1,2,4-[1,2,4]selena­diazolo[4,5-*a*]pyridin-4-ylium chloride, C_9_H_11_N_2_Se^+^·Cl^−^, in high yield (89%). The bifurcated Se⋯Cl^−^⋯H—Cl chalcogen-hydrogen-bonding inter­actions were analysed by DFT followed by a topological analysis of the electron-density distribution.

## 1. Chemical context

Recently, we discovered a novel cyclo­addition reaction between nitriles and 2-pyridyl­selenyl reagents (Artemjev *et al.*, 2023 ▸; Khrustalev *et al.*, 2021 ▸). Importantly, the reaction proceeds under mild conditions with high chemoselectivity and results in the formation of pyridinium-fused seleno­diazo­lium salts in high yields. The Se centre in these systems acts as a chalcogen bond donor and provides two *σ*-holes (Grudova *et al.*, 2022*a* ▸,*b* ▸). The 1,2,4-seleno­diazo­lium salts were shown to form supra­molecular dimers *via* four-center Se⋯*X* (*X* = Hal, N) chalcogen-bonding inter­actions (Grudova *et al.*, 2022*a* ▸,*b* ▸). In some instances, other types of supra­molecular organization were observed, depending on the nitrile employed in the cyclo­addition reaction (Grudova *et al.*, 2022*a* ▸,*b* ▸; Sapronov *et al.*, 2022 ▸, 2023 ▸; Artemjev *et al.*, 2022 ▸; Buslov *et al.*, 2021 ▸). 
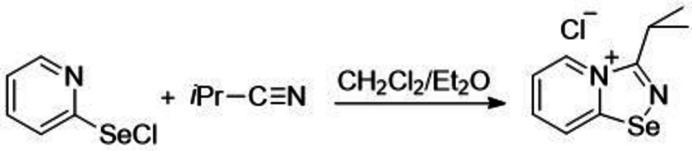


Here we report the preparation and structural characterization of a cationic pyridinium-fused 1,2,4-seleno­diazole, which was prepared *via* reaction of 2-pyridyl­selenenyl chloride with isobutyro­nitrile (reagent ratio of 1:1). The reaction was carried out under stirring at room temperature in CH_2_Cl_2_/Et_2_O over 24 h, which led to the formation of a white suspension. Isolation and purification gave a crystalline solid of the target compound in a yield of 89%.

## 2. Structural commentary

**Figure 1:**
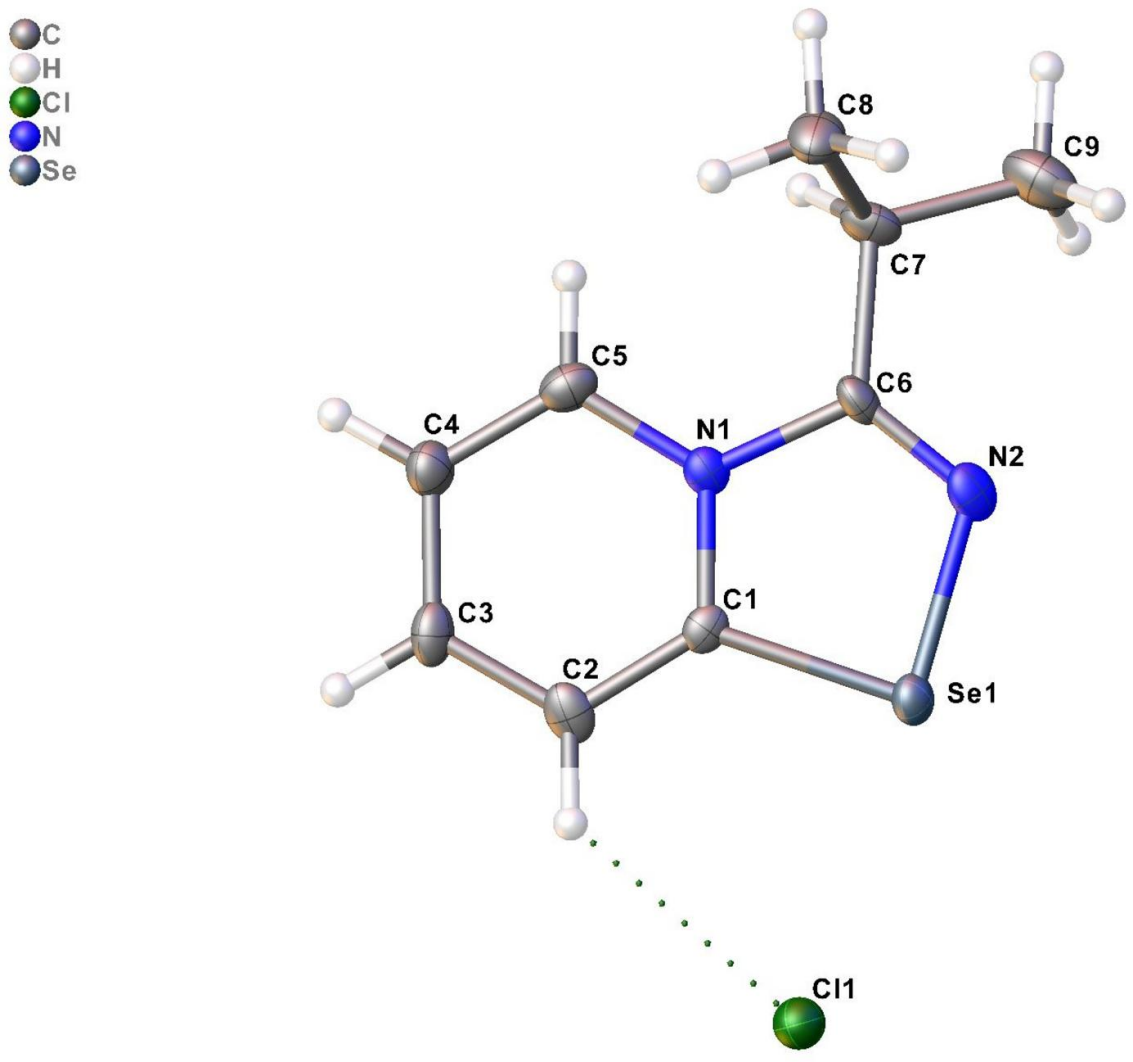
Mol­ecular structure of one of the four conformational isomers in the title compound.

The title compound (Fig. 1 ▸) crystallized in space group *P*2_1_/*c* with four cations and four Cl^−^ anions in the asymmetric unit. The four cations exhibit identical bond distances and angles, except for the dihedral angle of the isopropyl substituent [N—C—C—C torsion angles are in the range −15.9 (12) to 17.7 (11)°]. The 1,2,4-seleno­diazole fragments are almost planar (r.m.s.d. = 0.008–0.014 Å). The Se⋯Cl distances lie in the range 2.901 (3) – 2.956 (3) Å.

Inter­estingly, the novel 1,2,4-seleno­diazole did not form supra­molecular dimers via Se⋯N contacts.

## 3. Supra­molecular features and QTAIM analysis

**Figure 2:**
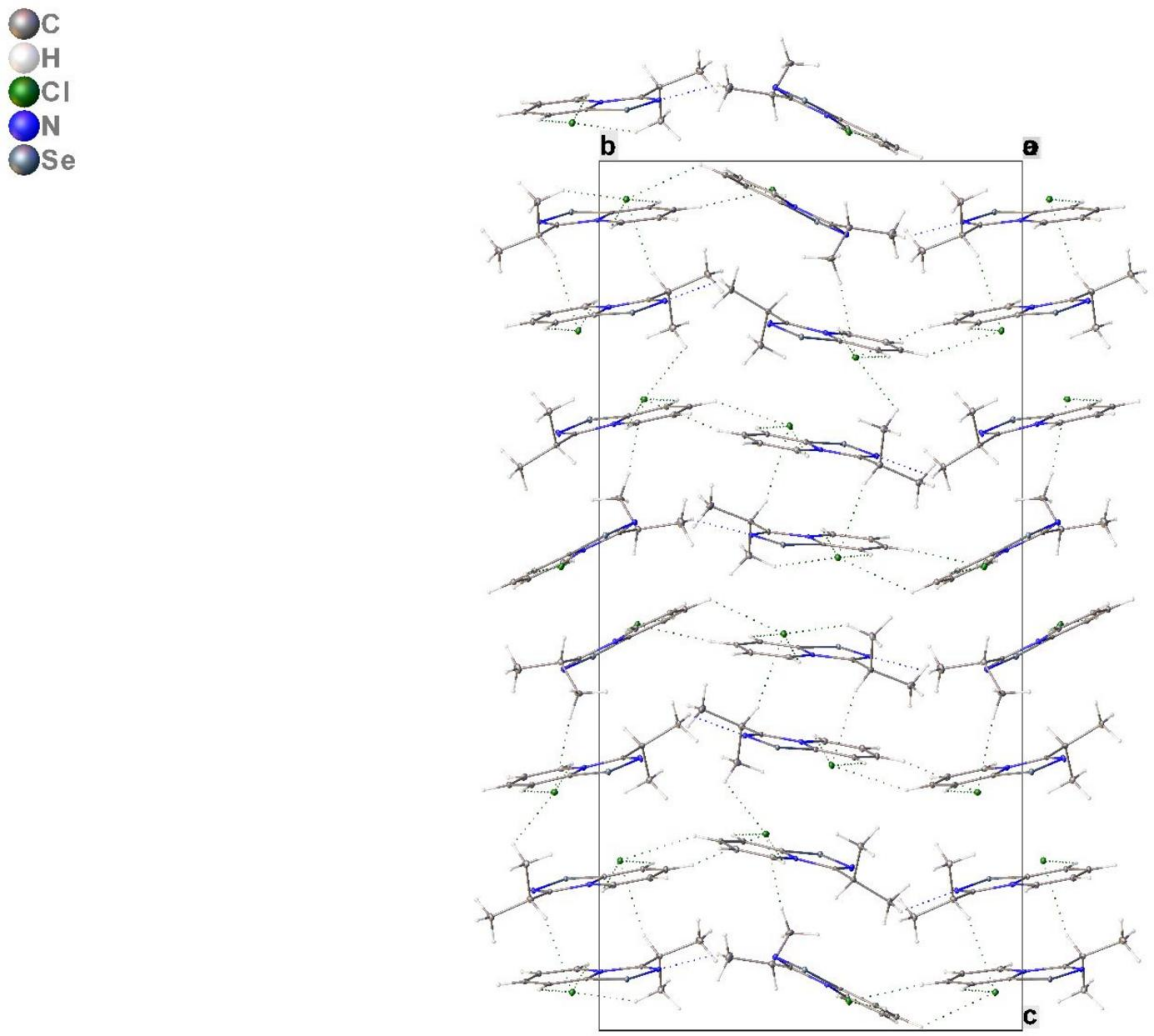
View along the *a* axis of the crystal packing of the title compound.

The crystal packing is shown in Fig. 2 ▸. The mol­ecules of the title compound are packed in layers parallel to the *ac* plane. Each row of 1,2,4-seleno­diazo­lium salts in the layer is located anti­parallel to the adjacent one. In addition to Se⋯Cl^−^ contacts (Table 1 ▸), the anions form C—H⋯Cl^−^ contacts (Table 2 ▸) that link the cations and anions both within the layers and between them.

**Figure 3:**
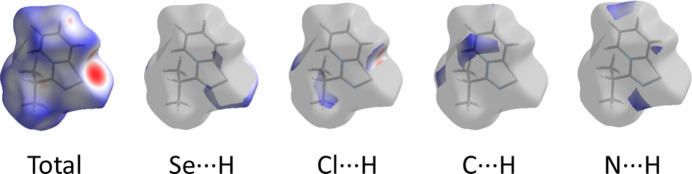
Total Hirshfeld surface mapped over *d*_norm_ and delineated into Se⋯H, Cl⋯H, C⋯H and N⋯H inter­actions.

A Hirshfeld surface analysis was performed to investigate which inter­atomic contacts make the largest contributions to the crystal packing. Fig. 3 ▸ shows the Hirshfeld surface mapped over *d*_norm_ where the region of the short inter­molecular Se⋯Cl contact is indicated by an intense red spot. The contributions of the different inter­atomic contacts to the Hirshfeld surface are H⋯H (47.0%), Se⋯H (10.5%), Cl⋯H (10.4%), C⋯H (10.1%), N⋯H (8.5%), Se⋯C (4.5%), Se⋯Cl (2.7%), Cl⋯C (1.8%), Se⋯N (1.6%), Cl⋯N (1.3%), N⋯C (1.0%), N⋯N (0.5%), and C⋯C (0.1%). Thus, the Hirshfeld surface analysis for the crystal structure reveals that crystal packing is determined primarily by inter­molecular contacts involving hydrogen atoms.

Inter­estingly, the title compound did not form supra­molecular dimers *via* Se⋯N contacts. To obtain a deeper understanding of the nature and qu­antify the strength of the bifurcated Se⋯Cl^−^⋯H—C chalcogen-hydrogen bonding in the title compound, single-point DFT calculations based on the experimental X-ray geometry were performed at the B97XD/6-311++G** level of theory using the dispersion-corrected hybrid functional ωB97XD using *GAUSSIAN09* (Frisch *et al.*, 2009 ▸) with the 6-311++G** basis sets used for all atoms, followed by a topological analysis of the electron-density distribution.

**Figure 4:**
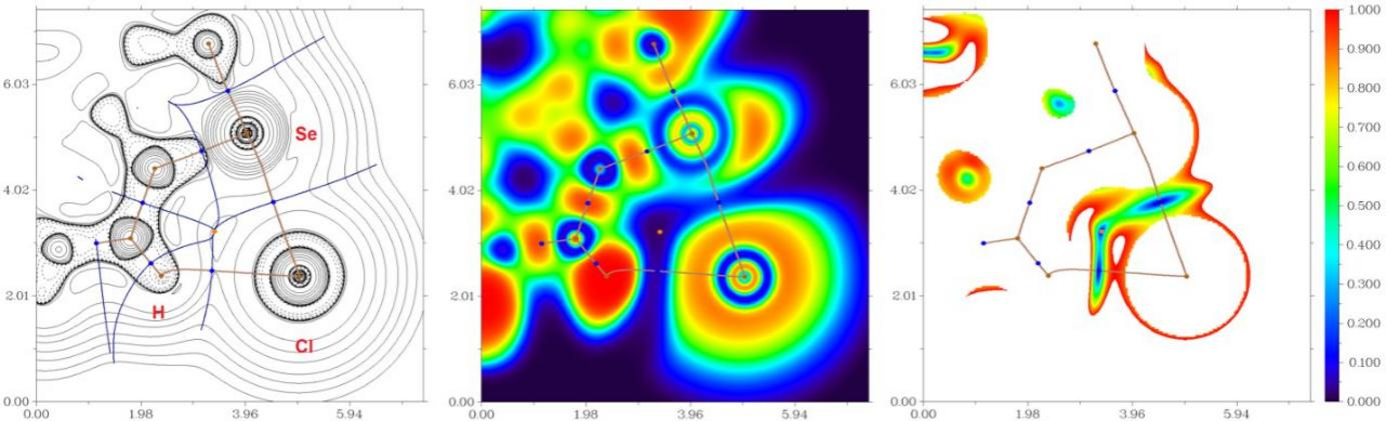
Contour line diagram of the Laplacian of electron density distribution ∇^2^r(**r**), bond paths, and selected zero-flux surfaces (left panel), visualization of electron localization function (ELF, center panel) and reduced density gradient (RDG, right panel) analyses for bifurcated chalcogen-hydrogen bonding Se⋯Cl^−^⋯H–C in sample (for Se⋯Cl^−^ 2.900 Å and C–H⋯Cl^−^ 2.609 Å). Bond critical points (3, −1) are shown in blue, nuclear critical points (3, −3) in pale brown, ring critical points (3, +1) in orange, bond paths are shown as pale-brown lines, length units are Å and the colour scale for the ELF and RDG maps is presented in a.u.

A QTAIM analysis of the model structure demonstrates the presence of bond critical points (3, −1) for short contacts Se⋯Cl^−^ and C—H⋯Cl^−^ in the formed 1,2,4-seleno­diazole (Table 3 ▸ and Fig. 4 ▸). The low magnitude of the electron density, positive values of the Laplacian of the electron density and zero or very close to zero values of energy density in these bond critical points (3, −1) and estimated strength for appropriate short contacts are typical for weak, purely non-covalent inter­actions (Espinosa *et al.*, 2002 ▸). Note that the nature of the discussed non-covalent contacts are similar to those weak inter­actions in closely related chemical systems (Grudova *et al.*, 2022*a* ▸,*b* ▸).

## 4. Database survey

A search in the Cambridge Structural Database (CSD, Version 5.43, update of Sep. 2022; Groom *et al.*, 2016 ▸) gave only 16 hits for 1,2,4-seleno­diazo­lium salts. These salts differ not only in the type of nitrile fragment [Me (EWEPUU; Khrustalev *et al.*, 2021 ▸), Ph (NAQDES; Buslov *et al.*, 2021 ▸), BrC_6_H_4_ (EWEQEF; Khrustalev *et al.*, 2021 ▸)], but also in the CF_3_COO^−^ anion (YEJXEU; Artemjev *et al.*, 2022 ▸), AuCl_4_^−^ (YEJXUK; Artemjev *et al.*, 2022 ▸), ReO_4_^−^ (YEJYAR; Artemjev *et al.*, 2022 ▸).

## 5. Synthesis and crystallization

**General remarks**. All manipulations were carried out in air and all reagents used in this study were obtained from commercial sources (Aldrich, TCI-Europe, Strem, ABCR). Commercially available solvents were purified by conventional methods and distilled immediately prior to use. NMR spectra were recorded on a Bruker Avance III (^1^H: 400 MHz); chemical shifts (*δ*) are given in ppm, coupling constants (*J*) in Hz. 2-Pyridyl­selenyl chloride was synthesized by our method (Artemjev *et al.*, 2023 ▸; Khrustalev *et al.*, 2021 ▸). Isobutyro­nitrile (81 µmol, 5.6 mg) was added to a suspension of 2-pyridyl­selenyl chloride (81 µmol, 15.5 mg) in CH_2_Cl_2_/Et_2_O (1/1, 4 mL), and the mixture was stirred at room temperature for 24 h. The formed colorless precipitate was filtered, washed with Et_2_O (3 × 1 mL) and dried under vacuum. Yield 18.8 mg (89%), colorless blocks. ^1^H NMR (400 MHz, chloro­form-*d*) *δ* 8.48 (*d*, *J* = 4.8 Hz, 1H), 7.83 (*d*, *J* = 7.9 Hz, 1H), 7.58 (*td*, *J* = 7.8 Hz, 1H), 7.12 (*td*, *J* = 7.5 Hz, 1H), 2.70 (*hept*, *J* = 7.0 Hz, 1H), 1.33 (*d*, *J* = 7.0 Hz, 6H). Crystals suitable for X-ray analysis were obtained by the slow evaporation of a CH_2_Cl_2_ solution.

## 6. Refinement

Crystal data, data collection and structure refinement details are summarized in Table 4 ▸. H atoms were positioned geom­etrically (C—H = 0.95–1.00 Å) and refined as riding with *U*_iso_(H) = 1.2–1.5*U*_eq_(C). The remaining positive and negative residual electron density close to the Se1, Se2, Se3 and Se4 atom positions (1.71 Å^−3^ at 0.94 Å from Se4, 1.67 Å^−3^ at 1.05 Å from Se2, 1.58 Å^−3^ at 1.03 Å from Se3, 1.54 Å^−3^ at 1.06 Å from Se4 and −1.53 Å^−3^ at 1.06 Å from Se4) suggests the possible presence of a small twin component as well.

## Supplementary Material

Crystal structure: contains datablock(s) I. DOI: 10.1107/S2056989024000938/ex2079sup1.cif

Structure factors: contains datablock(s) I. DOI: 10.1107/S2056989024000938/ex2079Isup2.hkl

Fingerprint plots. DOI: 10.1107/S2056989024000938/ex2079sup3.zip

CCDC reference: 2328546

Additional supporting information:  crystallographic information; 3D view; checkCIF report

## Figures and Tables

**Table 1:** Selected interatomic distances (Å)

Se1⋯Cl1	2.957 (4)	Se3⋯Cl3	2.934 (4)
Se1⋯N1	2.656 (8)	Se3⋯N5	2.661 (8)
Se2⋯Cl2	2.900 (4)	Se4⋯Cl4	2.920 (4)
Se2⋯N3	2.664 (7)	Se4⋯N7	2.658 (8)

**Table 2:** Hydrogen-bond geometry (Å, °)

*D*—H⋯*A*	*D*—H	H⋯*A*	*D*⋯*A*	*D*—H⋯*A*
C2—H2⋯Cl1	0.95	2.62	3.327 (10)	132
C3—H3⋯Cl2^i^	0.95	2.67	3.598 (9)	167
C5—H5⋯Cl1^ii^	0.95	2.67	3.395 (10)	133
C11—H11⋯Cl2	0.95	2.61	3.288 (10)	129
C14—H14⋯Cl2^ii^	0.95	2.47	3.310 (10)	147
C18—H18*C*⋯Cl4^iii^	0.98	2.73	3.687 (11)	167
C20—H20⋯Cl3	0.95	2.67	3.364 (10)	131
C23—H23⋯Cl3^iv^	0.95	2.73	3.418 (10)	130
C29—H29⋯Cl4	0.95	2.63	3.323 (10)	130
C30—H30⋯Cl3^v^	0.95	2.81	3.651 (9)	148
C32—H32⋯Cl4^ii^	0.95	2.76	3.452 (9)	131

Symmetry codes: (i) 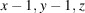 ; (ii) 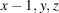 ; (iii) 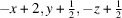 ; (iv) 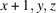 ; (v) 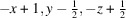 .

**Table 3:** Values of the density of all electrons ρ(**r**), Laplacian of electron density ∇^2ρ^(**r**) and appropriate λ_2_ eigenvalues, energy density – *H*_b_, potential energy density – *V*(**r**), Lagrangian kinetic energy – *G*(**r**), and electron localization function – *ELF* (a.u.) at the bond critical points (3, −1), corresponding to bifurcated chalcogen-hydrogen bonding Se⋯Cl^−^⋯H—C in the structure, and estimated strength for these inter­actions *E*_int_ ≃ –*V*(**r**)/2 (kcal mol^−1^) The Bondi (1966 ▸) van der Waals radii for the H, Se, and Cl atoms are 1.20, 1.90, and 1.75 Å, respectively.

Contact (Å)	ρ(**r**)	∇^2ρ^(**r**)	λ_2_	*H*_b_	*V*(**r**)	*G*(**r**)	*ELF*	*E*_int_
Se⋯Cl^−^ 2.900	0.027	0.060	−0.027	0.000	−0.015	0.015	0.170	4.7
C–H⋯Cl^−^ 2.609	0.012	0.043	−0.012	0.002	−0.006	0.008	0.045	1.9
Se⋯Cl^−^ 2.957	0.024	0.056	−0.024	0.001	−0.013	0.014	0.142	4.1
C–H⋯Cl^−^ 2.617	0.012	0.041	−0.012	0.002	−0.006	0.008	0.045	1.9
Se⋯Cl^−^ 2.934	0.025	0.058	−0.025	0.000	−0.014	0.014	0.147	4.4
C–H⋯Cl^−^ 2.667	0.011	0.037	−0.011	0.002	−0.005	0.007	0.041	1.6
Se⋯Cl^−^ 2.920	0.026	0.058	−0.026	0.000	−0.015	0.015	0.165	4.7
C–H⋯Cl^−^ 2.633	0.012	0.040	−0.012	0.002	−0.006	0.008	0.044	1.9

**Table 4:** Experimental details

Crystal data	
Chemical formula	C_9_H_11_N_2_Se^+^·Cl^−^
*M*_r_	261.61
Crystal system, space group	Monoclinic, *P*2_1_/*c*
Temperature (K)	100
*a*, *b*, *c* (Å)	9.054 (11), 15.015 (15), 30.93 (3)
β (°)	94.10 (3)
*V* (Å^3^)	4194 (8)
*Z*	16
Radiation type	Mo *K*α
μ (mm^−1^)	3.79
Crystal size (mm)	0.2 × 0.2 × 0.1

Data collection	
Diffractometer	Bruker D8 Venture
Absorption correction	Multi-scan (*SADABS*; Krause *et al.*, 2015 ▸)
*T*_min_, *T*_max_	0.499, 0.746
No. of measured, independent and observed [*I* > 2σ(*I*)] reflections	25216, 9604, 6328
*R*_int_	0.092
(sin θ/λ)_max_ (Å^−1^)	0.650

Refinement	
*R*[*F* ^2^ > 2σ(*F* ^2^)], *wR*(*F* ^2^), *S*	0.080, 0.192, 1.10
No. of reflections	9604
No. of parameters	477
H-atom treatment	H-atom parameters constrained
Δρ_max_, Δρ_min_ (e Å^−3^)	1.77, −1.49

Computer programs: *APEX2* and *SAINT* (Bruker, 2019 ▸), *SHELXT2014/5* (Sheldrick, 2015*a* ▸), *SHELXL2018/3* (Sheldrick, 2015*b* ▸) and *OLEX2* 1.5 (Dolomanov *et al.*, 2009 ▸).

## supplementary crystallographic information

### Crystal data

**Table:**

C_9_H_11_N_2_Se^+^·Cl^−^	*F*(000) = 2080
*M**_r_* = 261.61	*D*_x_ = 1.657 Mg m^−^^3^
Monoclinic, *P*2_1_/*c*	Mo *K*α radiation, λ = 0.71073 Å
*a* = 9.054 (11) Å	Cell parameters from 5157 reflections
*b* = 15.015 (15) Å	θ = 2.5–27.0°
*c* = 30.93 (3) Å	µ = 3.79 mm^−^^1^
β = 94.10 (3)°	*T* = 100 K
*V* = 4194 (8) Å^3^	Block, colourless
*Z* = 16	0.2 × 0.2 × 0.1 mm

### Data collection

**Table:**

Bruker D8 Venture diffractometer	6328 reflections with *I* > 2σ(*I*)
φ and ω scans	*R*_int_ = 0.092
Absorption correction: multi-scan (SADABS; Krause *et al.*, 2015)	θ_max_ = 27.5°, θ_min_ = 1.5°
*T*_min_ = 0.499, *T*_max_ = 0.746	*h* = −11→10
25216 measured reflections	*k* = −17→19
9604 independent reflections	*l* = −34→40

### Refinement

**Table:**

Refinement on *F*^2^	0 restraints
Least-squares matrix: full	Hydrogen site location: inferred from neighbouring sites
*R*[*F*^2^ > 2σ(*F*^2^)] = 0.080	H-atom parameters constrained
*wR*(*F*^2^) = 0.192	*w* = 1/[σ^2^(*F*_o_^2^) + (0.0572*P*)^2^ + 24.4862*P*] where *P* = (*F*_o_^2^ + 2*F*_c_^2^)/3
*S* = 1.10	(Δ/σ)_max_ < 0.001
9604 reflections	Δρ_max_ = 1.77 e Å^−^^3^
477 parameters	Δρ_min_ = −1.49 e Å^−^^3^

### Special details

**Table:**

Geometry. All esds (except the esd in the dihedral angle between two l.s. planes) are estimated using the full covariance matrix. The cell esds are taken into account individually in the estimation of esds in distances, angles and torsion angles; correlations between esds in cell parameters are only used when they are defined by crystal symmetry. An approximate (isotropic) treatment of cell esds is used for estimating esds involving l.s. planes.

### Fractional atomic coordinates and isotropic or equivalent isotropic displacement parameters (Å^2^)

**Table:**

	*x*	*y*	*z*	*U*_iso_*/*U*_eq_	
Se1	0.47831 (10)	0.56571 (5)	0.44129 (3)	0.0188 (2)	
N1	0.2029 (8)	0.5041 (4)	0.4321 (2)	0.0158 (14)	
N2	0.3169 (8)	0.6405 (4)	0.4298 (2)	0.0208 (15)	
C1	0.3415 (9)	0.4706 (5)	0.4407 (2)	0.0157 (16)	
C2	0.3662 (10)	0.3806 (5)	0.4468 (2)	0.0215 (19)	
H2	0.463568	0.358285	0.452949	0.026*	
C3	0.2475 (10)	0.3244 (5)	0.4437 (3)	0.0225 (19)	
H3	0.262519	0.262247	0.447472	0.027*	
C4	0.1014 (10)	0.3578 (5)	0.4348 (3)	0.0231 (19)	
H4	0.019521	0.318133	0.432535	0.028*	
C5	0.0794 (10)	0.4484 (5)	0.4294 (3)	0.0226 (18)	
H5	−0.017461	0.472085	0.423951	0.027*	
C6	0.1954 (10)	0.5998 (5)	0.4265 (3)	0.0182 (17)	
C7	0.0449 (10)	0.6424 (5)	0.4148 (3)	0.0228 (19)	
H7	−0.006834	0.606091	0.391248	0.027*	
C8	−0.0552 (10)	0.6457 (6)	0.4531 (3)	0.029 (2)	
H8A	−0.059140	0.586592	0.466403	0.043*	
H8B	−0.155223	0.664066	0.442583	0.043*	
H8C	−0.014747	0.688650	0.474718	0.043*	
C9	0.0689 (12)	0.7371 (6)	0.3968 (3)	0.037 (2)	
H9A	0.112941	0.775243	0.420018	0.055*	
H9B	−0.026482	0.762078	0.385810	0.055*	
H9C	0.135472	0.733964	0.373246	0.055*	
Se2	0.99777 (10)	0.98507 (5)	0.43039 (3)	0.0206 (2)	
N3	0.7271 (7)	1.0382 (4)	0.4473 (2)	0.0155 (14)	
N4	0.8305 (9)	0.9161 (4)	0.4154 (2)	0.0240 (16)	
C10	0.8686 (10)	1.0696 (5)	0.4521 (2)	0.0201 (17)	
C11	0.9007 (10)	1.1530 (5)	0.4712 (3)	0.0224 (18)	
H11	0.999354	1.174702	0.474969	0.027*	
C12	0.7821 (10)	1.2025 (5)	0.4842 (3)	0.0223 (18)	
H12	0.799270	1.260398	0.495885	0.027*	
C13	0.6357 (10)	1.1680 (5)	0.4805 (3)	0.0204 (18)	
H13	0.556089	1.201547	0.490621	0.024*	
C14	0.6109 (10)	1.0862 (5)	0.4623 (3)	0.0217 (18)	
H14	0.513573	1.062152	0.459912	0.026*	
C15	0.7132 (10)	0.9517 (5)	0.4276 (3)	0.0217 (18)	
C16	0.5614 (10)	0.9090 (5)	0.4235 (3)	0.0244 (19)	
H16	0.515032	0.917789	0.451551	0.029*	
C17	0.5773 (12)	0.8075 (6)	0.4161 (4)	0.043 (3)	
H17A	0.631217	0.797054	0.390244	0.065*	
H17B	0.478756	0.780391	0.412147	0.065*	
H17C	0.631904	0.780693	0.441382	0.065*	
C18	0.4603 (12)	0.9510 (6)	0.3879 (3)	0.034 (2)	
H18A	0.468793	1.015964	0.389582	0.051*	
H18B	0.357698	0.933378	0.391448	0.051*	
H18C	0.489245	0.930671	0.359565	0.051*	
Se3	0.26260 (10)	0.42116 (5)	0.32555 (3)	0.01773 (19)	
N5	0.5420 (8)	0.4775 (4)	0.3319 (2)	0.0199 (15)	
N6	0.4195 (9)	0.3446 (4)	0.3392 (2)	0.0226 (16)	
C19	0.4032 (9)	0.5118 (5)	0.3232 (3)	0.0178 (16)	
C20	0.3850 (10)	0.6028 (5)	0.3142 (3)	0.0198 (17)	
H20	0.288959	0.626641	0.307443	0.024*	
C21	0.5068 (10)	0.6569 (5)	0.3152 (3)	0.0232 (19)	
H21	0.495070	0.718889	0.309785	0.028*	
C22	0.6498 (11)	0.6212 (6)	0.3244 (3)	0.027 (2)	
H22	0.734293	0.658728	0.324740	0.033*	
C23	0.6658 (10)	0.5327 (5)	0.3327 (3)	0.0218 (18)	
H23	0.761829	0.508431	0.339056	0.026*	
C24	0.5435 (10)	0.3829 (5)	0.3405 (3)	0.0193 (17)	
C25	0.6916 (10)	0.3370 (6)	0.3495 (3)	0.026 (2)	
H25	0.754221	0.373184	0.370862	0.031*	
C26	0.7723 (11)	0.3267 (6)	0.3083 (3)	0.030 (2)	
H26A	0.707544	0.296467	0.286124	0.045*	
H26B	0.862308	0.291312	0.314442	0.045*	
H26C	0.798948	0.385609	0.297642	0.045*	
C27	0.6644 (12)	0.2436 (6)	0.3695 (3)	0.034 (2)	
H27A	0.595387	0.209768	0.349877	0.050*	
H27B	0.622106	0.250876	0.397530	0.050*	
H27C	0.758506	0.211384	0.373494	0.050*	
Se4	1.25396 (10)	0.52189 (5)	0.20254 (3)	0.0204 (2)	
N7	0.9756 (8)	0.4644 (4)	0.1977 (2)	0.0195 (15)	
N8	1.0966 (8)	0.5971 (4)	0.1871 (2)	0.0213 (15)	
C28	1.1139 (9)	0.4318 (5)	0.2066 (3)	0.0187 (17)	
C29	1.1317 (10)	0.3407 (5)	0.2175 (3)	0.0226 (19)	
H29	1.227441	0.316755	0.224650	0.027*	
C30	1.0089 (11)	0.2875 (5)	0.2175 (3)	0.025 (2)	
H30	1.020121	0.225748	0.223666	0.030*	
C31	0.8675 (10)	0.3229 (6)	0.2085 (3)	0.0253 (19)	
H31	0.782837	0.285610	0.209118	0.030*	
C32	0.8512 (9)	0.4109 (5)	0.1990 (3)	0.0201 (17)	
H32	0.755249	0.435634	0.193195	0.024*	
C33	0.9714 (10)	0.5569 (5)	0.1863 (2)	0.0205 (18)	
C34	0.8239 (10)	0.6012 (5)	0.1729 (3)	0.0220 (18)	
H34	0.766938	0.559546	0.152766	0.026*	
C35	0.7292 (10)	0.6186 (6)	0.2117 (3)	0.027 (2)	
H35A	0.783686	0.657512	0.232708	0.040*	
H35B	0.707740	0.561863	0.225694	0.040*	
H35C	0.636091	0.647310	0.201416	0.040*	
C36	0.8505 (10)	0.6869 (5)	0.1477 (3)	0.028 (2)	
H36A	0.755127	0.712260	0.136934	0.042*	
H36B	0.909601	0.673383	0.123245	0.042*	
H36C	0.903625	0.729918	0.166944	0.042*	
Cl1	0.7227 (2)	0.43621 (13)	0.45609 (7)	0.0235 (4)	
Cl2	1.2466 (2)	1.08940 (12)	0.46641 (7)	0.0237 (4)	
Cl3	0.0233 (2)	0.54927 (12)	0.30482 (7)	0.0218 (4)	
Cl4	1.4889 (2)	0.39388 (13)	0.22603 (7)	0.0227 (4)	

### Atomic displacement parameters (Å^2^)

**Table:**

	*U*^11^	*U*^22^	*U*^33^	*U*^12^	*U*^13^	*U*^23^
Se1	0.0212 (5)	0.0144 (4)	0.0206 (4)	−0.0026 (3)	−0.0009 (3)	−0.0022 (3)
N1	0.020 (4)	0.016 (3)	0.011 (3)	0.000 (3)	−0.001 (3)	−0.002 (2)
N2	0.030 (4)	0.016 (3)	0.016 (4)	0.000 (3)	−0.003 (3)	−0.005 (3)
C1	0.017 (4)	0.019 (4)	0.010 (4)	−0.003 (3)	0.000 (3)	−0.003 (3)
C2	0.033 (5)	0.019 (4)	0.012 (4)	0.000 (4)	−0.004 (4)	0.001 (3)
C3	0.031 (5)	0.018 (4)	0.018 (4)	−0.008 (4)	0.001 (4)	−0.003 (3)
C4	0.025 (5)	0.019 (4)	0.025 (5)	−0.003 (4)	−0.001 (4)	−0.003 (3)
C5	0.018 (5)	0.029 (4)	0.020 (4)	−0.001 (4)	−0.001 (3)	−0.002 (3)
C6	0.024 (5)	0.011 (3)	0.019 (4)	0.003 (3)	−0.003 (3)	−0.001 (3)
C7	0.022 (5)	0.025 (4)	0.020 (4)	0.008 (4)	−0.004 (4)	0.001 (3)
C8	0.023 (5)	0.025 (4)	0.039 (6)	0.002 (4)	0.009 (4)	−0.005 (4)
C9	0.042 (7)	0.036 (5)	0.032 (6)	0.015 (5)	0.001 (5)	0.007 (4)
Se2	0.0193 (5)	0.0179 (4)	0.0244 (5)	0.0017 (3)	−0.0002 (3)	−0.0009 (3)
N3	0.013 (3)	0.016 (3)	0.018 (4)	0.004 (3)	−0.001 (3)	−0.002 (2)
N4	0.030 (4)	0.019 (3)	0.023 (4)	0.003 (3)	−0.003 (3)	−0.003 (3)
C10	0.027 (5)	0.022 (4)	0.010 (4)	0.001 (4)	−0.004 (3)	0.004 (3)
C11	0.026 (5)	0.023 (4)	0.018 (4)	−0.007 (4)	−0.006 (4)	0.002 (3)
C12	0.023 (5)	0.020 (4)	0.023 (5)	−0.001 (4)	0.000 (4)	0.000 (3)
C13	0.020 (5)	0.021 (4)	0.021 (4)	0.006 (3)	0.000 (3)	−0.001 (3)
C14	0.018 (4)	0.018 (4)	0.029 (5)	0.002 (3)	0.001 (4)	0.003 (3)
C15	0.032 (5)	0.017 (4)	0.015 (4)	−0.002 (4)	−0.007 (4)	0.002 (3)
C16	0.021 (5)	0.026 (4)	0.025 (5)	−0.005 (4)	0.001 (4)	−0.001 (3)
C17	0.039 (7)	0.032 (5)	0.058 (8)	−0.010 (5)	−0.003 (5)	−0.009 (5)
C18	0.038 (6)	0.041 (5)	0.022 (5)	−0.005 (5)	−0.006 (4)	−0.007 (4)
Se3	0.0200 (4)	0.0133 (4)	0.0197 (4)	−0.0013 (3)	−0.0001 (3)	−0.0003 (3)
N5	0.021 (4)	0.023 (3)	0.016 (4)	0.002 (3)	−0.002 (3)	−0.003 (3)
N6	0.032 (5)	0.016 (3)	0.020 (4)	0.000 (3)	0.002 (3)	0.001 (3)
C19	0.018 (4)	0.017 (4)	0.019 (4)	0.005 (3)	0.003 (3)	0.000 (3)
C20	0.024 (5)	0.013 (4)	0.023 (4)	0.001 (3)	−0.001 (3)	0.000 (3)
C21	0.025 (5)	0.014 (4)	0.030 (5)	0.001 (3)	−0.002 (4)	−0.001 (3)
C22	0.029 (5)	0.025 (4)	0.028 (5)	−0.010 (4)	−0.001 (4)	−0.001 (4)
C23	0.015 (4)	0.024 (4)	0.027 (5)	0.000 (3)	0.001 (3)	−0.002 (3)
C24	0.027 (5)	0.015 (4)	0.016 (4)	0.005 (3)	−0.003 (3)	−0.001 (3)
C25	0.023 (5)	0.026 (4)	0.028 (5)	0.002 (4)	−0.004 (4)	0.003 (3)
C26	0.027 (6)	0.032 (5)	0.031 (5)	0.011 (4)	0.003 (4)	−0.002 (4)
C27	0.039 (6)	0.026 (5)	0.035 (6)	0.010 (4)	0.004 (5)	0.009 (4)
Se4	0.0192 (5)	0.0170 (4)	0.0246 (5)	−0.0007 (3)	−0.0022 (3)	0.0011 (3)
N7	0.024 (4)	0.015 (3)	0.018 (4)	−0.003 (3)	−0.003 (3)	−0.003 (3)
N8	0.018 (4)	0.015 (3)	0.031 (4)	0.005 (3)	0.000 (3)	0.007 (3)
C28	0.019 (4)	0.019 (4)	0.018 (4)	0.001 (3)	−0.003 (3)	−0.005 (3)
C29	0.024 (5)	0.018 (4)	0.023 (5)	0.003 (3)	−0.011 (4)	0.000 (3)
C30	0.034 (6)	0.019 (4)	0.022 (5)	0.003 (4)	−0.007 (4)	0.001 (3)
C31	0.021 (5)	0.026 (4)	0.028 (5)	−0.009 (4)	−0.001 (4)	−0.004 (3)
C32	0.013 (4)	0.025 (4)	0.023 (5)	0.000 (3)	−0.001 (3)	−0.006 (3)
C33	0.036 (5)	0.012 (4)	0.012 (4)	−0.007 (3)	−0.002 (4)	0.002 (3)
C34	0.025 (5)	0.023 (4)	0.016 (4)	0.003 (4)	−0.007 (4)	−0.001 (3)
C35	0.022 (5)	0.024 (4)	0.034 (5)	0.004 (4)	0.000 (4)	−0.004 (4)
C36	0.026 (5)	0.018 (4)	0.038 (6)	0.008 (4)	−0.008 (4)	0.004 (3)
Cl1	0.0217 (11)	0.0213 (10)	0.0273 (11)	0.0000 (8)	0.0012 (8)	−0.0019 (8)
Cl2	0.0184 (11)	0.0191 (9)	0.0332 (12)	0.0000 (8)	−0.0020 (9)	0.0056 (8)
Cl3	0.0191 (11)	0.0183 (9)	0.0278 (11)	0.0021 (8)	−0.0001 (8)	−0.0026 (7)
Cl4	0.0197 (11)	0.0264 (10)	0.0216 (11)	0.0025 (8)	−0.0005 (8)	0.0008 (8)

### Geometric parameters (Å, º)

**Table:**

Se1—N2	1.857 (7)	Se3—N6	1.853 (7)
Se1—C1	1.890 (8)	Se3—C19	1.868 (8)
N1—C1	1.360 (10)	N5—C19	1.367 (10)
N1—C5	1.394 (10)	N5—C23	1.393 (11)
N1—C6	1.448 (9)	N5—C24	1.444 (10)
N2—C6	1.256 (11)	N6—C24	1.260 (11)
C1—C2	1.381 (11)	C19—C20	1.402 (10)
C2—H2	0.9500	C20—H20	0.9500
C2—C3	1.364 (12)	C20—C21	1.369 (12)
C3—H3	0.9500	C21—H21	0.9500
C3—C4	1.422 (12)	C21—C22	1.411 (12)
C4—H4	0.9500	C22—H22	0.9500
C4—C5	1.383 (11)	C22—C23	1.359 (11)
C5—H5	0.9500	C23—H23	0.9500
C6—C7	1.526 (11)	C24—C25	1.516 (12)
C7—H7	1.0000	C25—H25	1.0000
C7—C8	1.542 (12)	C25—C26	1.523 (12)
C7—C9	1.549 (12)	C25—C27	1.559 (12)
C8—H8A	0.9800	C26—H26A	0.9800
C8—H8B	0.9800	C26—H26B	0.9800
C8—H8C	0.9800	C26—H26C	0.9800
C9—H9A	0.9800	C27—H27A	0.9800
C9—H9B	0.9800	C27—H27B	0.9800
C9—H9C	0.9800	C27—H27C	0.9800
Se2—N4	1.866 (8)	Se4—N8	1.854 (7)
Se2—C10	1.882 (8)	Se4—C28	1.865 (8)
N3—C10	1.363 (11)	N7—C28	1.354 (11)
N3—C14	1.383 (10)	N7—C32	1.386 (10)
N3—C15	1.437 (10)	N7—C33	1.433 (9)
N4—C15	1.270 (11)	N8—C33	1.283 (11)
C10—C11	1.407 (11)	C28—C29	1.415 (11)
C11—H11	0.9500	C29—H29	0.9500
C11—C12	1.389 (12)	C29—C30	1.369 (12)
C12—H12	0.9500	C30—H30	0.9500
C12—C13	1.420 (12)	C30—C31	1.396 (12)
C13—H13	0.9500	C31—H31	0.9500
C13—C14	1.364 (11)	C31—C32	1.361 (11)
C14—H14	0.9500	C32—H32	0.9500
C15—C16	1.513 (12)	C33—C34	1.522 (12)
C16—H16	1.0000	C34—H34	1.0000
C16—C17	1.550 (12)	C34—C35	1.546 (12)
C16—C18	1.518 (12)	C34—C36	1.533 (11)
C17—H17A	0.9800	C35—H35A	0.9800
C17—H17B	0.9800	C35—H35B	0.9800
C17—H17C	0.9800	C35—H35C	0.9800
C18—H18A	0.9800	C36—H36A	0.9800
C18—H18B	0.9800	C36—H36B	0.9800
C18—H18C	0.9800	C36—H36C	0.9800
			
Se1···Cl1	2.957 (4)	Se3···Cl3	2.934 (4)
Se1···N1	2.656 (8)	Se3···N5	2.661 (8)
Se2···Cl2	2.900 (4)	Se4···Cl4	2.920 (4)
Se2···N3	2.664 (7)	Se4···N7	2.658 (8)
			
N2—Se1—C1	87.0 (3)	N6—Se3—C19	87.0 (3)
C1—N1—C5	121.0 (7)	C19—N5—C23	120.4 (7)
C1—N1—C6	115.1 (7)	C19—N5—C24	113.8 (7)
C5—N1—C6	123.9 (7)	C23—N5—C24	125.8 (7)
C6—N2—Se1	113.2 (5)	C24—N6—Se3	113.0 (5)
N1—C1—Se1	108.5 (5)	N5—C19—Se3	109.7 (5)
N1—C1—C2	121.8 (7)	N5—C19—C20	120.0 (8)
C2—C1—Se1	129.7 (7)	C20—C19—Se3	130.3 (7)
C1—C2—H2	120.8	C19—C20—H20	120.3
C3—C2—C1	118.5 (8)	C21—C20—C19	119.4 (8)
C3—C2—H2	120.8	C21—C20—H20	120.3
C2—C3—H3	119.6	C20—C21—H21	119.9
C2—C3—C4	120.8 (8)	C20—C21—C22	120.3 (7)
C4—C3—H3	119.6	C22—C21—H21	119.9
C3—C4—H4	120.2	C21—C22—H22	120.2
C5—C4—C3	119.6 (8)	C23—C22—C21	119.6 (8)
C5—C4—H4	120.2	C23—C22—H22	120.2
N1—C5—H5	120.8	N5—C23—H23	119.9
C4—C5—N1	118.3 (8)	C22—C23—N5	120.2 (8)
C4—C5—H5	120.8	C22—C23—H23	119.9
N1—C6—C7	118.5 (7)	N5—C24—C25	118.5 (7)
N2—C6—N1	116.1 (7)	N6—C24—N5	116.5 (7)
N2—C6—C7	125.3 (7)	N6—C24—C25	124.9 (7)
C6—C7—H7	108.0	C24—C25—H25	109.0
C6—C7—C8	113.3 (7)	C24—C25—C26	111.3 (7)
C6—C7—C9	108.9 (7)	C24—C25—C27	108.6 (8)
C8—C7—H7	108.0	C26—C25—H25	109.0
C8—C7—C9	110.6 (7)	C26—C25—C27	109.8 (7)
C9—C7—H7	108.0	C27—C25—H25	109.0
C7—C8—H8A	109.5	C25—C26—H26A	109.5
C7—C8—H8B	109.5	C25—C26—H26B	109.5
C7—C8—H8C	109.5	C25—C26—H26C	109.5
H8A—C8—H8B	109.5	H26A—C26—H26B	109.5
H8A—C8—H8C	109.5	H26A—C26—H26C	109.5
H8B—C8—H8C	109.5	H26B—C26—H26C	109.5
C7—C9—H9A	109.5	C25—C27—H27A	109.5
C7—C9—H9B	109.5	C25—C27—H27B	109.5
C7—C9—H9C	109.5	C25—C27—H27C	109.5
H9A—C9—H9B	109.5	H27A—C27—H27B	109.5
H9A—C9—H9C	109.5	H27A—C27—H27C	109.5
H9B—C9—H9C	109.5	H27B—C27—H27C	109.5
N4—Se2—C10	87.0 (4)	N8—Se4—C28	86.9 (3)
C10—N3—C14	121.0 (7)	C28—N7—C32	121.7 (7)
C10—N3—C15	114.3 (7)	C28—N7—C33	114.2 (7)
C14—N3—C15	124.6 (7)	C32—N7—C33	124.1 (7)
C15—N4—Se2	112.0 (6)	C33—N8—Se4	112.3 (5)
N3—C10—Se2	109.3 (5)	N7—C28—Se4	110.3 (5)
N3—C10—C11	121.2 (8)	N7—C28—C29	119.1 (7)
C11—C10—Se2	129.4 (7)	C29—C28—Se4	130.6 (7)
C10—C11—H11	121.4	C28—C29—H29	120.5
C12—C11—C10	117.2 (8)	C30—C29—C28	119.1 (8)
C12—C11—H11	121.4	C30—C29—H29	120.5
C11—C12—H12	119.4	C29—C30—H30	119.6
C11—C12—C13	121.2 (8)	C29—C30—C31	120.7 (8)
C13—C12—H12	119.4	C31—C30—H30	119.6
C12—C13—H13	120.4	C30—C31—H31	120.1
C14—C13—C12	119.1 (8)	C32—C31—C30	119.8 (8)
C14—C13—H13	120.4	C32—C31—H31	120.1
N3—C14—H14	120.0	N7—C32—H32	120.2
C13—C14—N3	120.1 (8)	C31—C32—N7	119.6 (8)
C13—C14—H14	120.0	C31—C32—H32	120.2
N3—C15—C16	118.1 (7)	N7—C33—C34	119.8 (7)
N4—C15—N3	117.1 (8)	N8—C33—N7	116.3 (8)
N4—C15—C16	124.8 (7)	N8—C33—C34	123.8 (7)
C15—C16—H16	107.8	C33—C34—H34	107.5
C15—C16—C17	109.6 (8)	C33—C34—C35	112.8 (7)
C15—C16—C18	112.5 (7)	C33—C34—C36	109.8 (7)
C17—C16—H16	107.8	C35—C34—H34	107.5
C18—C16—H16	107.8	C36—C34—H34	107.5
C18—C16—C17	111.1 (8)	C36—C34—C35	111.6 (7)
C16—C17—H17A	109.5	C34—C35—H35A	109.5
C16—C17—H17B	109.5	C34—C35—H35B	109.5
C16—C17—H17C	109.5	C34—C35—H35C	109.5
H17A—C17—H17B	109.5	H35A—C35—H35B	109.5
H17A—C17—H17C	109.5	H35A—C35—H35C	109.5
H17B—C17—H17C	109.5	H35B—C35—H35C	109.5
C16—C18—H18A	109.5	C34—C36—H36A	109.5
C16—C18—H18B	109.5	C34—C36—H36B	109.5
C16—C18—H18C	109.5	C34—C36—H36C	109.5
H18A—C18—H18B	109.5	H36A—C36—H36B	109.5
H18A—C18—H18C	109.5	H36A—C36—H36C	109.5
H18B—C18—H18C	109.5	H36B—C36—H36C	109.5
			
Se1—N2—C6—N1	−0.2 (9)	Se3—N6—C24—N5	0.9 (9)
Se1—N2—C6—C7	−176.5 (6)	Se3—N6—C24—C25	−178.1 (6)
Se1—C1—C2—C3	−178.3 (6)	Se3—C19—C20—C21	178.7 (7)
N1—C1—C2—C3	0.3 (12)	N5—C19—C20—C21	−1.4 (12)
N1—C6—C7—C8	74.0 (9)	N5—C24—C25—C26	−73.8 (9)
N1—C6—C7—C9	−162.4 (7)	N5—C24—C25—C27	165.2 (7)
N2—Se1—C1—N1	0.8 (5)	N6—Se3—C19—N5	0.4 (6)
N2—Se1—C1—C2	179.5 (8)	N6—Se3—C19—C20	−179.7 (8)
N2—C6—C7—C8	−109.8 (9)	N6—C24—C25—C26	105.2 (10)
N2—C6—C7—C9	13.8 (11)	N6—C24—C25—C27	−15.9 (12)
C1—Se1—N2—C6	−0.3 (6)	C19—Se3—N6—C24	−0.7 (6)
C1—N1—C5—C4	−1.5 (11)	C19—N5—C23—C22	−0.5 (12)
C1—N1—C6—N2	0.9 (10)	C19—N5—C24—N6	−0.6 (10)
C1—N1—C6—C7	177.5 (7)	C19—N5—C24—C25	178.5 (7)
C1—C2—C3—C4	−0.5 (12)	C19—C20—C21—C22	1.3 (13)
C2—C3—C4—C5	−0.2 (13)	C20—C21—C22—C23	−0.8 (13)
C3—C4—C5—N1	1.2 (12)	C21—C22—C23—N5	0.4 (13)
C5—N1—C1—Se1	179.5 (6)	C23—N5—C19—Se3	−179.0 (6)
C5—N1—C1—C2	0.7 (11)	C23—N5—C19—C20	1.1 (11)
C5—N1—C6—N2	−179.7 (7)	C23—N5—C24—N6	178.4 (7)
C5—N1—C6—C7	−3.2 (11)	C23—N5—C24—C25	−2.6 (12)
C6—N1—C1—Se1	−1.1 (8)	C24—N5—C19—Se3	0.0 (8)
C6—N1—C1—C2	−180.0 (7)	C24—N5—C19—C20	−179.9 (7)
C6—N1—C5—C4	179.3 (7)	C24—N5—C23—C22	−179.4 (8)
Se2—N4—C15—N3	4.0 (9)	Se4—N8—C33—N7	0.8 (9)
Se2—N4—C15—C16	−175.0 (6)	Se4—N8—C33—C34	−177.4 (6)
Se2—C10—C11—C12	179.6 (6)	Se4—C28—C29—C30	−179.0 (7)
N3—C10—C11—C12	−0.9 (11)	N7—C28—C29—C30	1.8 (12)
N3—C15—C16—C17	−161.4 (7)	N7—C33—C34—C35	74.5 (9)
N3—C15—C16—C18	74.5 (10)	N7—C33—C34—C36	−160.3 (7)
N4—Se2—C10—N3	1.6 (5)	N8—Se4—C28—N7	−1.2 (6)
N4—Se2—C10—C11	−178.9 (8)	N8—Se4—C28—C29	179.6 (8)
N4—C15—C16—C17	17.6 (12)	N8—C33—C34—C35	−107.3 (9)
N4—C15—C16—C18	−106.5 (10)	N8—C33—C34—C36	17.9 (11)
C10—Se2—N4—C15	−3.2 (6)	C28—Se4—N8—C33	0.2 (6)
C10—N3—C14—C13	2.9 (12)	C28—N7—C32—C31	−1.3 (12)
C10—N3—C15—N4	−2.8 (10)	C28—N7—C33—N8	−1.9 (10)
C10—N3—C15—C16	176.3 (7)	C28—N7—C33—C34	176.5 (7)
C10—C11—C12—C13	3.2 (12)	C28—C29—C30—C31	−2.4 (13)
C11—C12—C13—C14	−2.5 (12)	C29—C30—C31—C32	1.1 (13)
C12—C13—C14—N3	−0.7 (12)	C30—C31—C32—N7	0.8 (13)
C14—N3—C10—Se2	177.4 (6)	C32—N7—C28—Se4	−179.3 (6)
C14—N3—C10—C11	−2.1 (11)	C32—N7—C28—C29	0.0 (11)
C14—N3—C15—N4	180.0 (7)	C32—N7—C33—N8	179.3 (7)
C14—N3—C15—C16	−1.0 (11)	C32—N7—C33—C34	−2.3 (11)
C15—N3—C10—Se2	0.1 (8)	C33—N7—C28—Se4	1.9 (8)
C15—N3—C10—C11	−179.5 (7)	C33—N7—C28—C29	−178.8 (7)
C15—N3—C14—C13	−180.0 (7)	C33—N7—C32—C31	177.4 (7)

### Hydrogen-bond geometry (Å, º)

**Table:**

*D*—H···*A*	*D*—H	H···*A*	*D*···*A*	*D*—H···*A*
C2—H2···Cl1	0.95	2.62	3.327 (10)	132
C3—H3···Cl2^i^	0.95	2.67	3.598 (9)	167
C5—H5···Cl1^ii^	0.95	2.67	3.395 (10)	133
C11—H11···Cl2	0.95	2.61	3.288 (10)	129
C14—H14···Cl2^ii^	0.95	2.47	3.310 (10)	147
C18—H18*C*···Cl4^iii^	0.98	2.73	3.687 (11)	167
C20—H20···Cl3	0.95	2.67	3.364 (10)	131
C23—H23···Cl3^iv^	0.95	2.73	3.418 (10)	130
C29—H29···Cl4	0.95	2.63	3.323 (10)	130
C30—H30···Cl3^v^	0.95	2.81	3.651 (9)	148
C32—H32···Cl4^ii^	0.95	2.76	3.452 (9)	131

Symmetry codes: (i) *x*−1, *y*−1, *z*; (ii) *x*−1, *y*, *z*; (iii) −*x*+2, *y*+1/2, −*z*+1/2; (iv) *x*+1, *y*, *z*; (v) −*x*+1, *y*−1/2, −*z*+1/2.
