# Supplementary figures and images for: Synthesis and crystal structure of the adduct between 2-pyridyl­selenyl chloride and isobutyro­nitrile
Source: Acta Crystallogr E Crystallogr Commun. 2024 Feb 6;80(Pt 3):247–51. doi: 10.1107/S2056989024000938 (PMC10915658; doi:10.1107/S2056989024000938)

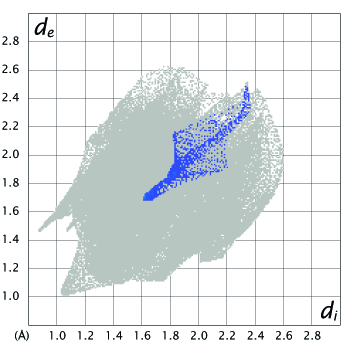

Supplement: Supplementary file 3 [file e-80-00247-sup3.zip › Cl···C.tif]

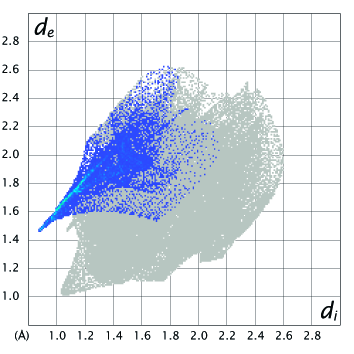

Supplement: Supplementary file 3 [file e-80-00247-sup3.zip › Cl···H.tif]

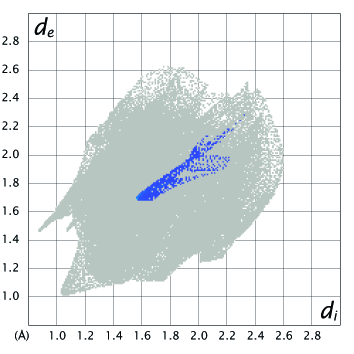

Supplement: Supplementary file 3 [file e-80-00247-sup3.zip › Cl···N.tif]

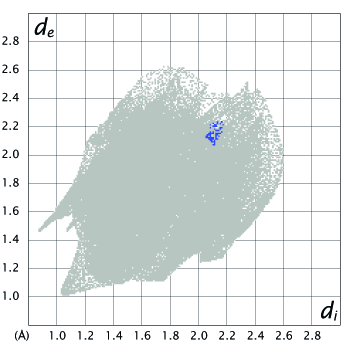

Supplement: Supplementary file 3 [file e-80-00247-sup3.zip › C···C.tif]

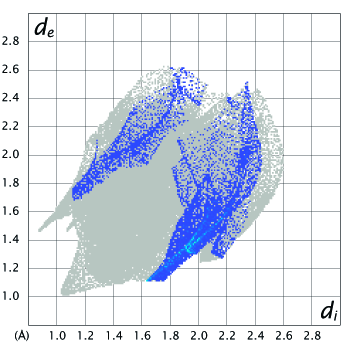

Supplement: Supplementary file 3 [file e-80-00247-sup3.zip › C···H.tif]

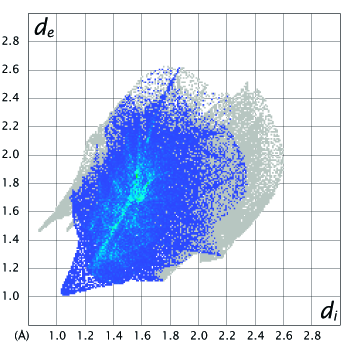

Supplement: Supplementary file 3 [file e-80-00247-sup3.zip › H···H.tif]

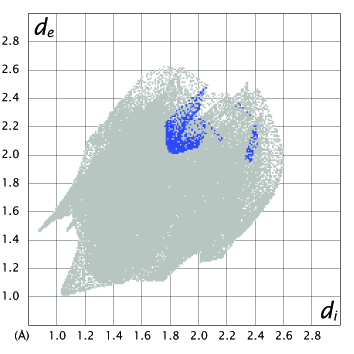

Supplement: Supplementary file 3 [file e-80-00247-sup3.zip › N···C.tif]

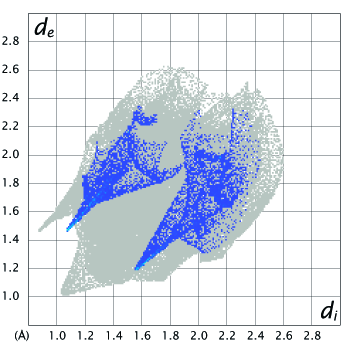

Supplement: Supplementary file 3 [file e-80-00247-sup3.zip › N···H.tif]

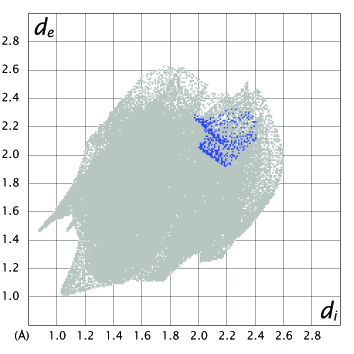

Supplement: Supplementary file 3 [file e-80-00247-sup3.zip › N···N.tif]

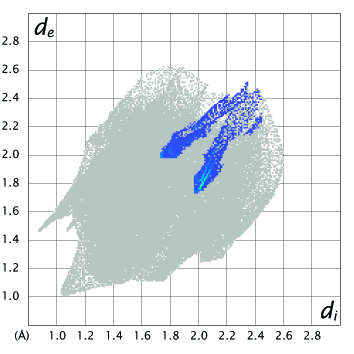

Supplement: Supplementary file 3 [file e-80-00247-sup3.zip › Se···C.tif]

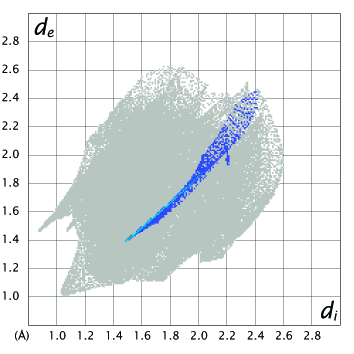

Supplement: Supplementary file 3 [file e-80-00247-sup3.zip › Se···Cl.tif]

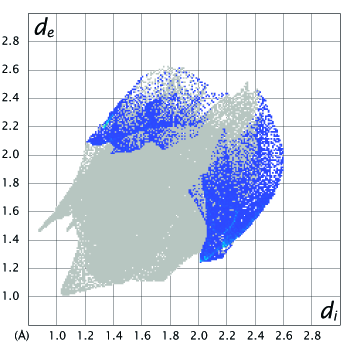

Supplement: Supplementary file 3 [file e-80-00247-sup3.zip › Se···H.tif]

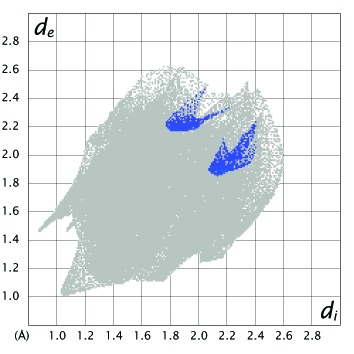

Supplement: Supplementary file 3 [file e-80-00247-sup3.zip › Se···N.tif]

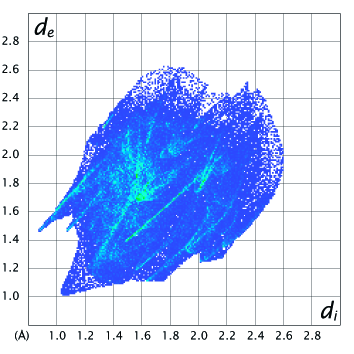

Supplement: Supplementary file 3 [file e-80-00247-sup3.zip › total.tif]
